# Chest Radiography Pearls in Select Adult Congenital Heart Disease

**DOI:** 10.3390/jpm14040397

**Published:** 2024-04-09

**Authors:** William A. Schiavone, David S. Majdalany

**Affiliations:** 1 Cleveland Clinic Heart and Vascular Institute, Cleveland, OH 44120, USA; schiavw@gmail.com; 2 Department of Cardiovascular Diseases, Mayo Clinic, Phoenix, AZ 85054, USA

**Keywords:** chest X-ray (CXR), Adult Congenital Heart Disease (ACHD)

## Abstract

Congenital heart disease in adult patients (ACHD) includes individuals with native anatomic deformities and those who have benefited from corrective, ameliorative, or interventional heart and vascular interventions. Congenital heart disease is the most common birth defect, although with interventions most survive into adulthood. Newborns and children with complex congenital heart diseases that feature cyanosis fail to thrive, and once this is identified, heart failure can promptly undergo diagnostic evaluations and treatment. However, patients with simple congenital heart disease and subtle clinical signs and symptoms may escape diagnosis until adulthood or experience changes in their cardiac hemodynamics and physiology in settings such as pregnancy or newly diagnosed arrhythmias. The chest X-ray (CXR) is the most common X-ray among all radiological procedures. Individual features or a constellation of features on a CXR are often present in patients who have congenital heart disease. The ability to recognize these CXR features is a valuable skill for making the diagnosis of ACHD and for following these patients as they age, and can complement echocardiographic findings. When used well to diagnose ACHD, the CXR will be the sharpest arrow in the quiver.

## 1. Introduction

As a clinician interpreting a CXR, it is important to note if the image is well centered. This is determined when the upper dorsal vertebral spinous processes are equidistant from the heads of the clavicles and these spinous processes are visible in the center of the trachea. The depth of inspiration influences the transcardiac dimension. A good inspiration will permit at least 10 posterior ribs to be visualized through the aerated lung. A normal heart size will have a transcardiac dimension that is </= 50% of the transthoracic dimension. This is known as the cardiothoracic (C-T) ratio. The preferred CXR is obtained with the patient standing erect with the anterior chest apposing the CXR plate and the X-ray beam directed from posterior to anterior. In this posteroanterior (PA) image the C-T ratio is most accurate. When an anteroposterior (AP) CXR image is obtained, the C-T ratio is larger. This is due to the shadow of the heart splaying out as the X-ray beam enters the chest anteriorly and exposes the X-ray plate as more distant from the heart than when imaged in the PA fashion. The scapulae are visible, and seen to be underlying the upper lung fields in an AP CXR. When a lateral (Lat) CXR image is demonstrated, its pertinent features will be described. The C-T ratio from a PA CXR is a simple, reproducible and independent marker of disease severity and outcome in ACHD [1].

## 2. Discussion of Select Adult Congenital Heart Disease Cases

Each ACHD diagnosis will be presented first, describing its anatomy and physiology. Patient symptoms and pertinent physical examination findings will be discussed. Then the CXR(s) of an actual patient will be presented, highlighting important CXR features of this ACHD.

### 2.1. Congenital Complete Absence of the Left Portion of the Parietal Pericardium (CCALPPP)

#### Anatomy and Physiology of Congenital Complete Absence of the Left Portion of the Parietal Pericardium

**Figure 1:**
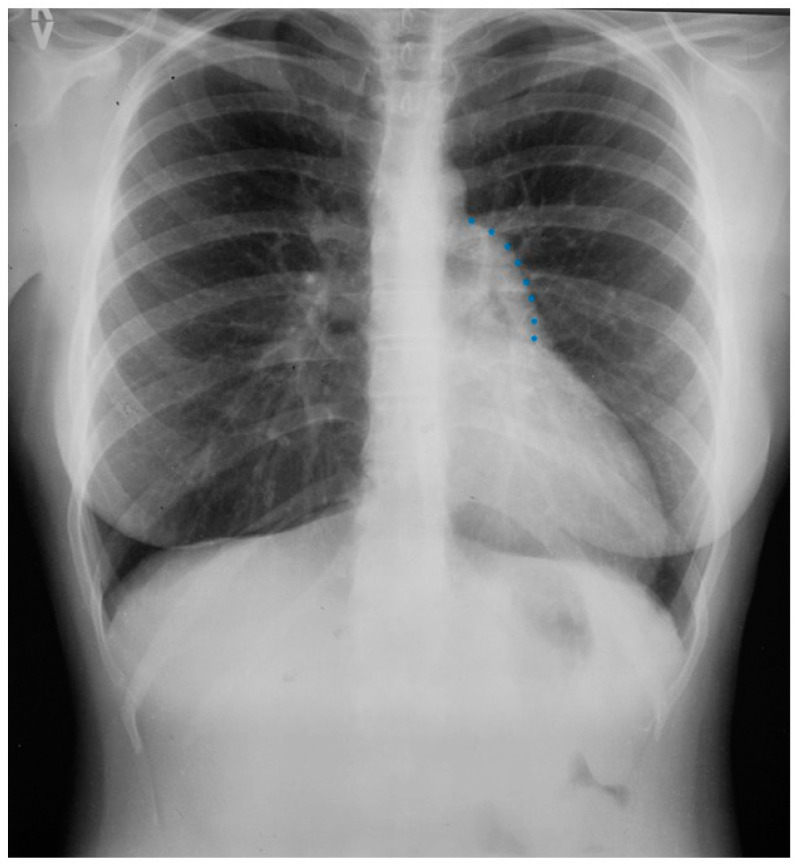
Posteroanterior chest X-ray in congenital complete absence of the left portion of the parietal pericardium: A 30-year-old female was referred to a cardiologist because of an unusual chest X-ray done as part of an executive physical. She was asymptomatic and her vital signs and heart sounds were normal and there was no heart murmur or arrhythmia. The image is well centered, there is a good inspiration and the C-T ratio is <0.5. The heart is shifted into the left chest and the right heart border is obscured because it overlies the dorsal vertebral bodies. The pulmonary artery segment (outlined with blue dots) is enlarged but the pulmonary vascularity is normal, making left to right shunting unlikely. Echocardiography and Cardiac MRI confirmed she had CCALPPP. She was reassured. No treatment was necessary.

The heart is normally anchored in the center of the chest by the pericardium. Between the visceral and the parietal pericardia is 25–50 mL of pericardial fluid, which reduces friction during cardiac contraction. When the left portion of the parietal pericardium is completely absent, the heart shifts to the left hemithorax and the patient often presents with paroxysmal, stabbing chest pain that is nonexertional [2]. This pain has been attributed to episodic torsion of the great vessels because the heart is unmoored. Physical examination usually reveals cardiac apical displacement by palpation. Heart sounds, including splitting of S2, are normal. There is often a soft ejection systolic murmur that is best heard at the left sternal border. Although CCALPPP might be an uncomfortable congenital heart disease, all 10 patients studied by Gatzoulis et al. [2] were alive and well more than 10 years after diagnosis. In cases where the left pericardial defect is partial, especially when there is a foramen-type partial absence of the left pericardium, the left atrium can herniate into the defect and strangulate the heart. When recognized, this partial defect must be remedied. The incidence of congenital absence of the pericardium, both complete and partial, is very rare. As many cases are asymptomatic, the majority of cases are recognized unwittingly at autopsy or during thoracotomy, when addressing a different problem [2]. The PA CXR in CCALPPP demonstrates levoposition of the heart, loss of the right heart border as it overlies the vertebral bodies, and prominence of the pulmonary artery segment (Figure 1). 

### 2.2. Pericardial Cysts

#### Anatomy and Physiology of Pericardial Cysts

**Figure 2:**
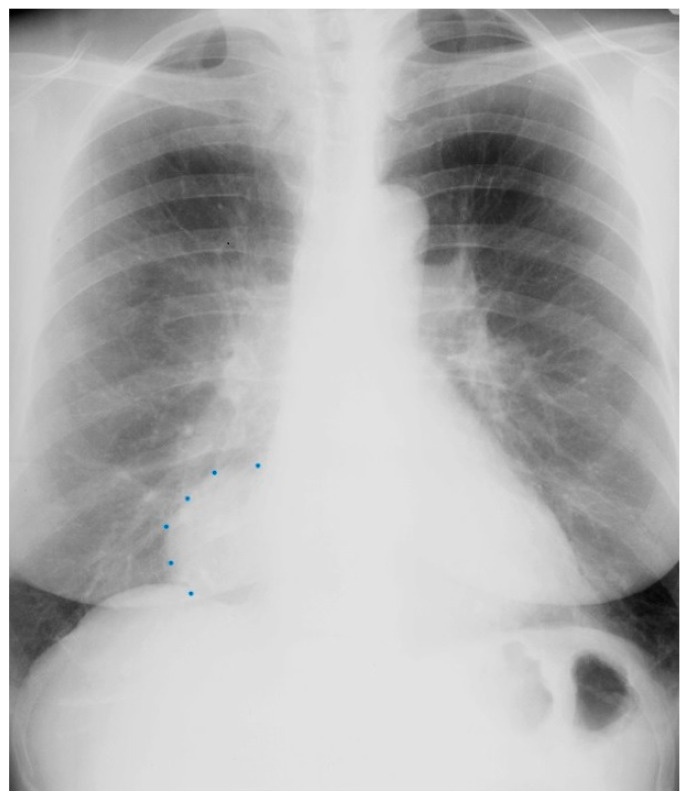
Posteroanterior chest X-ray of Pericardial Cyst: This is a chest X-ray of a 40-year-old female with a pericardial cyst. The film is well centered and there is a good inspiration. The cardiothoracic ratio is normal. Just above the aortic knob the manubrium sterni is well focused. It is shaped like a trapezoid and articulates with the clavicular heads. The lungs and pulmonary vascularity appear to be normal. There is a mass at the right cardiophrenic angle that obscures the lower right-heart border. This heart border obscuration is called the silhouette sign and is present because the mass is anterior in the chest and adjacent to the heart. This mass is a pericardial cyst (blue dots). The pericardial cyst is slightly more radiolucent than the heart because it is a thin-walled structure that is filled with pericardial fluid. A pericardial cyst is also known as a spring water cyst. As she was asymptomatic and there was no physical examination evidence of cardiovascular compromise, she received no treatment and was periodically observed.

**Figure 3:**
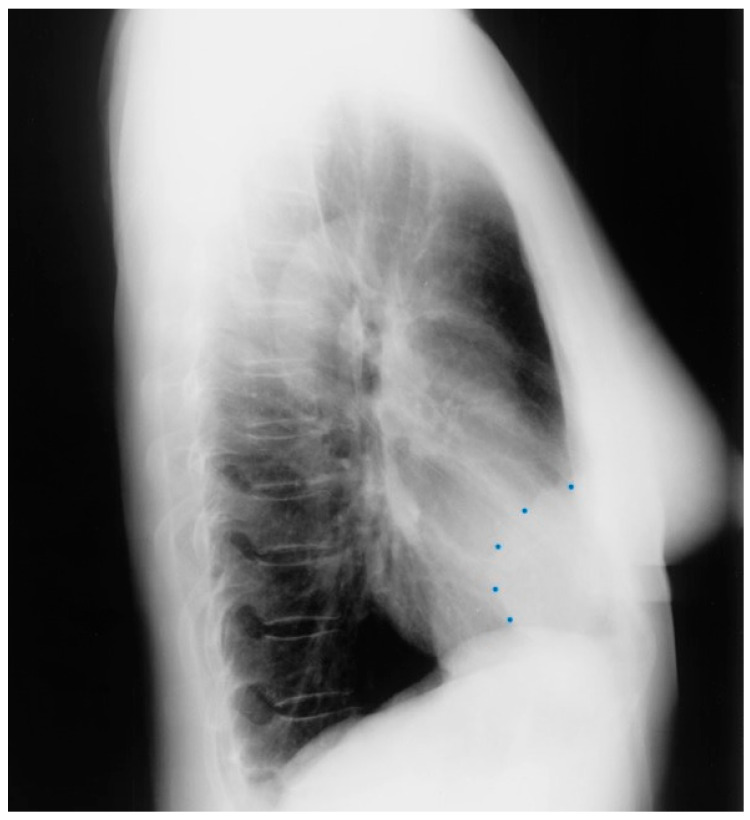
Lateral chest X-ray of a pericardial cyst: This is the lateral chest X-ray of the same-40 year-old female with a right cardiophrenic angle pericardial cyst. This image demonstrates that this pericardial cyst (blue dots) is located anteriorly in the chest and adjacent to the heart, and explains the silhouette sign on the posteroanterior chest X-ray. In this image, because the cyst and the heart are superimposed, their radiodensities are summed and the cyst appears denser than the rest of the heart that it does not overlie.

Pericardial cysts are the third most common type of mediastinal mass [3]. Pericardial cysts are benign congenital anomalies of the middle mediastinum. Formation of a pericardial cyst is due to abnormal fusion of the mesenchymal lacunae during embryogenesis and occur in ~1 in 100,000 people. A pericardial cyst might also be a result of trauma [4]. As pericardial cysts are usually asymptomatic, they are most often incidentally found by CXR or by transthoracic echocardiography. A pericardial cyst is commonly located at the right cardiophrenic angle (Figure 2 and Figure 3). Other less common locations are the left costophrenic angle and anterior/posterior mediastinum. Symptoms that have been attributed to pericardial cysts include dyspnea, and palpitations. Albeit a rare occurrence, when a pericardial cyst ruptures it can cause pleuritic chest pain [5]. 

### 2.3. Coarctation of the Aorta

#### Anatomy and Physiology of Coarctation of the Aorta

**Figure 4:**
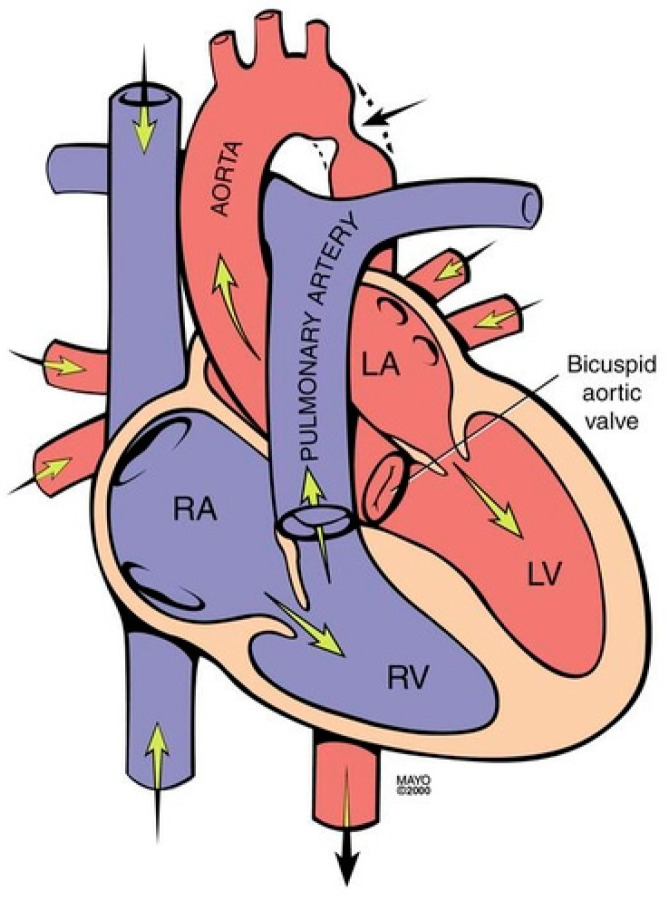
Coarctation of the aorta revealing narrowing in the region of the ligamentum arteriosum adjacent to the origin of the left subclavian artery (black arrow). It is frequently associated with a bicuspid valve. RA: right atrium; RV: right ventricle; LA: left atrium; LV: left ventricle.

**Figure 5:**
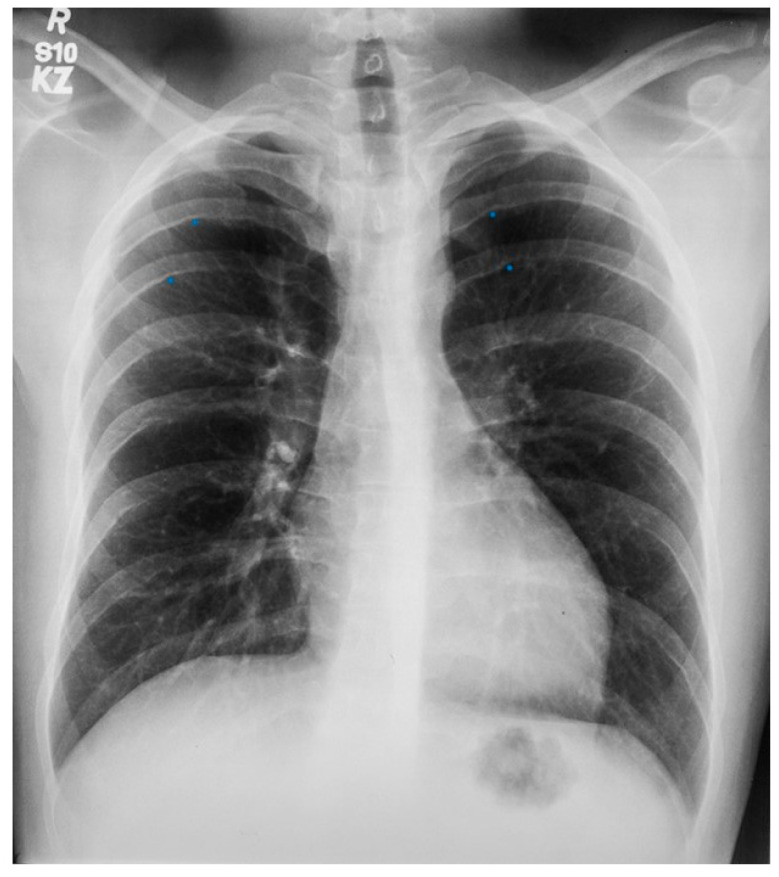
Posteroanterior chest X-ray of coarctation of the aorta: This is the chest X-ray of a 21-year-old male college student who presented for evaluation of hypertension. He spent most of his time studying, because he did not excel in sports due to cramping in his legs when he would run. The blood pressure was 170/80 mmHg in the right upper extremity and there was a marked brachial artery–femoral artery delay when simultaneously palpated. There was a grade 2–3/6 decrescendo diastolic murmur of aortic regurgitation. In the left upper posterior chest there was a long systolic murmur across the coarctation. This chest X-ray is well centered and there is a good inspiration. The cardiothoracic ratio was at the upper limits of normal and there is straightening of the left heart border, due to left ventricular volume overload from severe aortic regurgitation and left ventricular hypertrophy from his coarctation. There is rib notching of the undersides of ribs 4–8 bilaterally (indicated by four blue dots) from enlarged and coiled intercostal arteries carrying collateral blood flow. There is no three-sign, perhaps because this is not an anteroposterior film. He went on to have surgical repair of the coarctation via left lateral thoracotomy and then aortic valve replacement via median sternotomy for a bicuspid aortic valve with severe regurgitation. His blood pressure and his heart size both decreased and his tolerance for exertion improved.

Coarctation of the aorta (CoA) is a common congenital heart and vascular defect that accounts for 5–8% of all congenital cardiac defects [6]. Discrete CoA (Figure 4) consists of a short segment of narrowing in the region of the ligamentum arteriosum adjacent to the origin of the left subclavian artery [7]. Extensive collateral vessels often arise proximal to the obstruction and deliver blood distal to the obstruction sufficient for bodily function, albeit to a limited extent. Bicuspid aortic valve (BAV) and aortopathy are often found in patients who have CoA. When a patient is first diagnosed with CoA as an adult, the usual presentation is systemic arterial hypertension. When asked if they can keep up with their peers in a foot race, patients with CoA will respond “not in the long run”. This is due to claudication. Physical examination of the patient with CoA will show prominent neck pulsations and hypertension, often higher in the right arm than the left arm. Simultaneous palpation of the right brachial artery pulse and the right femoral artery pulse demonstrates a delayed femoral pulse. This is because the route is longer from the heart through the collaterals to the femoral artery than from the heart to the brachial artery. The systolic blood pressure (BP) is also decreased in the lower extremities compared to the right upper extremity. Auscultation in the upper chest, especially the upper posterior chest, will show a long systolic murmur (extending into diastole) across the coarctation. When a BAV is present, an ejection click, a systolic murmur of aortic stenosis and/or a diastolic murmur of aortic regurgitation may be present. The AP CXR in CoA may show a prominent curvilinear shadow along the mid-right sternal border that represents a dilated ascending aorta [6]. A “3-sign” adjacent to the area beneath the transverse arch and above the main pulmonary artery silhouette is often produced by an indentation at the site of the coarctation. Notching of the underside of ribs 3–9 is often found in CoA (Figure 5) and is due to compression of the ribs by enlarged and coiled intercostal arteries that serve as collaterals from the internal thoracic arteries to the descending aorta. The C-T ratio can be >0.5 when there is severe aortic regurgitation due to BAV. There often is straightening of the left heart border due to left ventricular hypertrophy from the increased systemic workload of hypertension from the CoA.

### 2.4. Scimitar Syndrome

#### Anatomy and Physiology of Scimitar Syndrome

**Figure 6:**
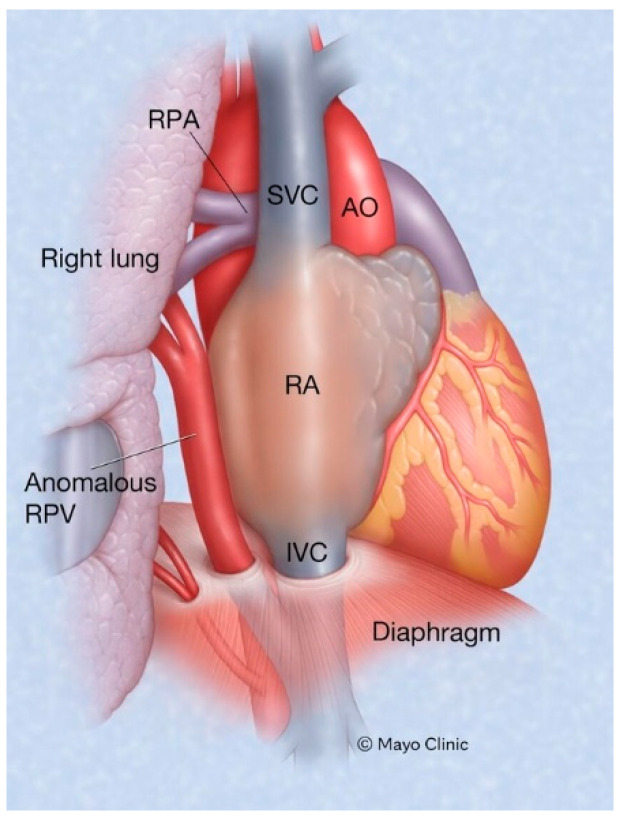
Scimitar syndrome: Part of all the right pulmonary venous return emptying into the inferior vena cava is a hallmark of Scimitar syndrome. AO: aorta; IVC: inferior vena cava; RA: right atrium; RPA: right pulmonary artery; SVC: superior vena cava.

**Figure 7:**
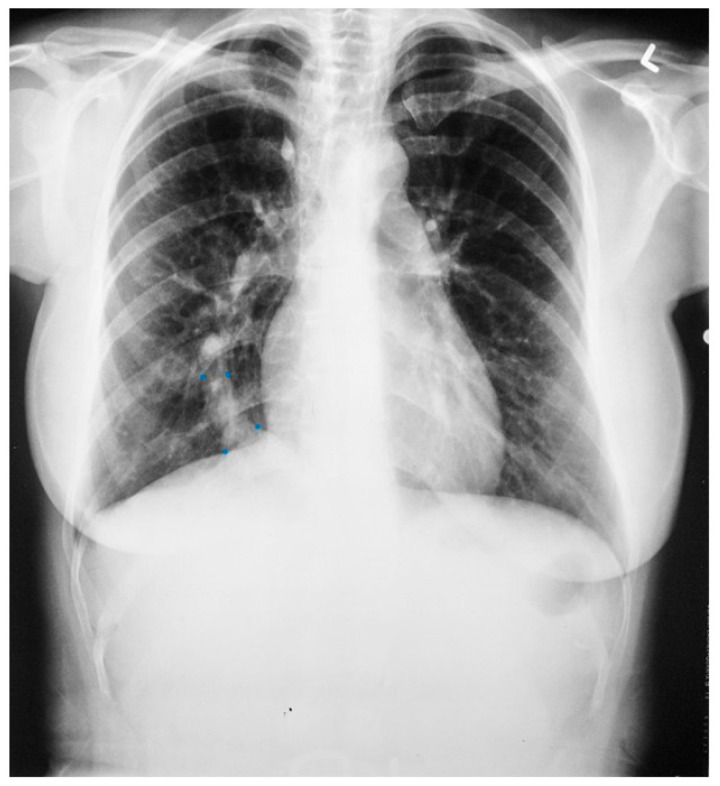
Posteroanterior chest X-ray of Scimitar syndrome: This is a 40-year-old asymptomatic female who had a chest X-ray as part of a routine physical examination. The film is well centered and there is a good inspiration. The ratio is normal. The right lung is normal in size, but note at the right lung apex there is an azygos fissure with a laterally-displaced azygos vein. There is a discrete scimitar paralleling the right heart border. Notice how the caliber of the scimitar increases as it courses inferiorly. The right-to-left distance between the upper two blue dots that straddle the scimitar is smaller than the distance between the lower two scimitar-straddling blue dots. The fact that the pulmonary arteries are not plethoric indicates that the left-to-right shunt (right lower pulmonary vein to inferior vena cava) is not large. Cardiac auscultation corroborated this. There was no systolic pulmonary flow murmur and S2 demonstrated physiological splitting that was not widely split or fixed. There was mild right ventricular volume overload by echocardiography. Her exercise capacity was normal. She received no treatment and periodic F/U was scheduled.

Scimitar syndrome is a rare congenital cardiopulmonary abnormality that has been reported in 3–6% of patients with partial anomalous pulmonary venous drainage. In these patients, some of the pulmonary veins (usually veins draining the right lower lobe and less often also the right middle lobe or the entire right lung) drain into the upper part of the inferior vena cava (IVC), either above or below the diaphragm (Figure 6). The right lung may be hypoplastic and then receives its blood supply from the thoracic or abdominal aorta [8]. The shadow of the anomalous pulmonary vein (Figure 7) that enlarges from the right hilum as it courses towards the right hemidiaphragm and drains into the IVC has the same shape as a Turkish sword called a scimitar. Scimitar syndrome gets its name from the appearance of the PA CXR. It looks like there is a scimitar positioned to the right of the heart pointing to the right hemidiaphragm. While scimitar syndrome with hypoplastic lung and other congenital malformations is almost always diagnosed in childhood, when only partial anomalous pulmonary venous drainage is present, the patient may be asymptomatic and not recognized until a CXR is done in adulthood.

### 2.5. Sinus Venosus Atrial Septal Defect

#### Anatomy and Physiology of Sinus Venosus Atrial Septal Defect

**Figure 8:**
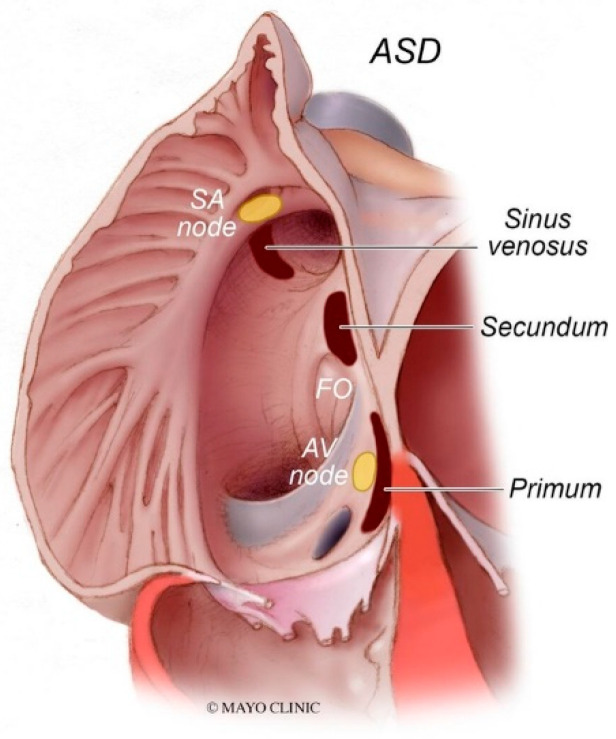
Types of atrial septal defects: Sinus venosus atrial septal defect is typically in the superior and posterior aspect of the inter-atrial septum and is commonly associated with partial anomalous pulmonary venous return. Secundum atrial septal defects, which are the most common type of atrial septal defects, occur in the middle of the inter-atrial septum. Primum atrial septal defects occur in the inferior aspect of the inter-atrial septal and are commonly associated with cleft atrio-ventricular valves. AV node: atrioventricular node; FO: fossa ovalis; SA node: sinoatrial node.

**Figure 9:**
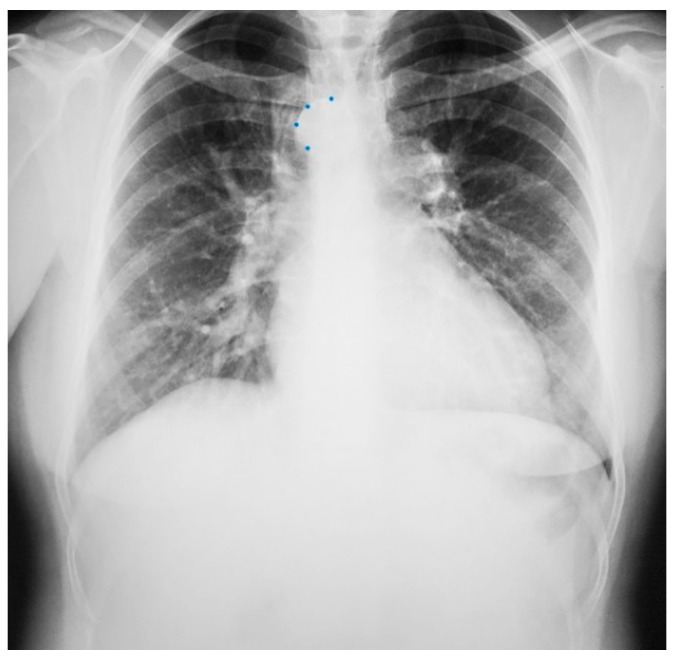
Posteroanterior chest X-ray of sinus venosus atrial septal defect. This chest X-ray is of a 24-year-old female with a sinus venosus atrial septal defect and anomalous right upper pulmonary venous drainage to the superior vena cava. The image is well centered and there is a good inspiration. The cardiothoracic ratio is increased, the pulmonary artery segment is increased and the pulmonary vascularity is plethoric. Also note that there is a right-sided aortic arch (blue dots). This allows the enlarged pulmonary trunk to be better visualized because the thoracic aorta does not obscure it. Because she had dyspnea, fatigue and exercise intolerance she underwent surgical repair of the ASD and redirection of the right upper pulmonary vein to the left atrium. Her postoperative CXR showed decrease in C-T ratio and pulmonary vascularity. Her symptoms resolved.

Sinus venosus atrial septal defect (SVASD) is the least common of the three main types of atrial septal defect (ostium secundum, ostium primum and sinus venosus). SVASD is located highest in the atrial septum and is also known as Defect Superior to Fossa Ovalis. This defect is in the region where the superior vena cava (SVC) joins the right atrium (Figure 8) and is often accompanied by anomalous termination of the right upper pulmonary vein(s) in the SVC near its right atrial junction or in the right atrium near the SVC junction [9]. As is seen in all ASDs, the resulting left-to-right shunting in SVASD increases the right atrial size and right ventricular size. A large shunt will present with dyspnea, fatigue, exercise intolerance, palpitations and/or syncope [7]. Physical examination may include a left parasternal heave, a pulmonary systolic flow murmur and fixed splitting of the second heart sound. The CXR may show right ventricular enlargement, right atrial enlargement, a prominent pulmonary artery segment and increased pulmonary vascularity (Figure 9). In the presence of a large left-to-right shunt, the volume of blood that is pumped to the lungs may be two or more times the volume pumped systemically. This is reflected on the PA CXR by the size of the pulmonary artery segment exceeding that of the aorta.

### 2.6. Ostium Secundum Atrial Septal Defect

#### Anatomy and Physiology of Ostium Secundum Atrial Septal Defect

**Figure 10:**
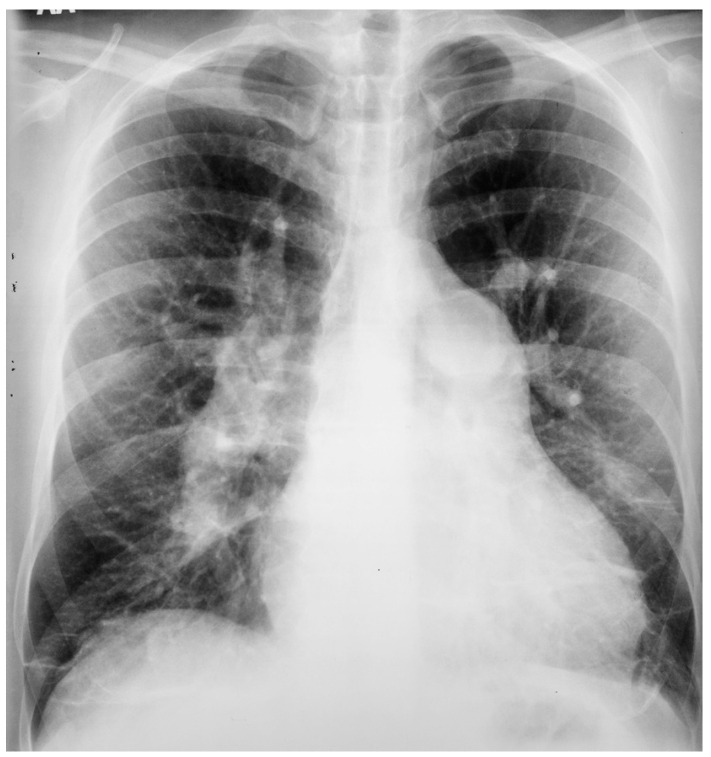
Posteroanterior chest X-ray of ostium secundum atrial septal defect (OSASD): This is a chest X-ray of a 46-year-old male with an uncorrected OSASD. The image is slightly rotated. There is a good inspiration. The cardiothoracic ratio is increased. The pulmonary trunk is enlarged and there is a lateral rim of calcification. Compared to the enlarged central pulmonary circulation the more peripheral pulmonary vessels are much smaller (called pruning), suggesting pulmonary vascular obstructive disease. The lower right heart border demonstrates right atrial enlargement. The aortic knob is smaller than the pulmonary trunk, indicating a significant left to right shunt.

An atrial septal defect (ASD) is among the most common congenital heart defects. A persistent ASD in the region of the fossa ovalis is called ostium secundum atrial septal defect (OSASD) and it comprises 75% of all ASDs. An OSASD (Figure 8) is different from a Patent Foramen Ovale (PFO), which is an interatrial communication in which the septum primum covering the fossa ovalis overlaps the superior limbic band of the septum secundum [10]. A PFO is usually closed when the pressure in the left atrium exceeds the pressure in the right atrium, but it opens when right atrial pressure exceeds left atrial pressure. A native OSASD results in left-to-right shunting across the defect, right ventricular volume overload and pulmonary overcirculation. A large left-to-right shunt may result in flow-related pulmonary artery hypertension and pulmonary vascular obstructive disease may develop in adult years [10]. The initial adult presentation of OSASD is most often characterized by symptoms of dyspnea and palpitations. Physical examination includes a precordial lift, a systolic pulmonary flow murmur and, usually, fixed splitting of S2. The PA CXR may show right atrial enlargement, a prominent pulmonary artery segment and increased pulmonary vascularity (Figure 10). In the presence of pulmonary vascular obstructive disease the CXR will demonstrate pruning of peripheral pulmonary arteries.

### 2.7. Congenitally Corrected Transposition of the Great Arteries

#### Anatomy and Physiology of Congenitally Corrected Transposition of the Great Arteries

**Figure 11:**
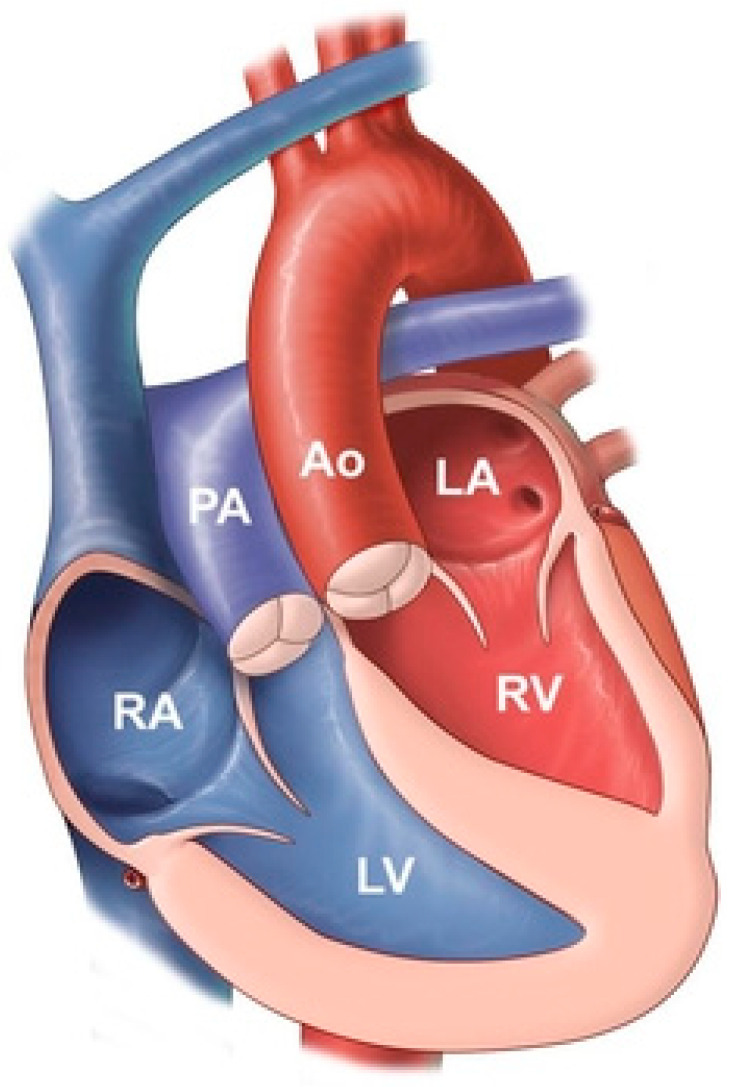
Congenitally corrected transposition of the great arteries: it consists of atrio-ventricular discordance and ventricular-arterial discordance. The right atrium (RA) is connected to the left ventricle (LV), which pumps deoxygenated blood into the pulmonary artery (PA). The left atrium (LA) is connected to the right ventricle, which pumps oxygenated blood into the aorta (Ao).

**Figure 12:**
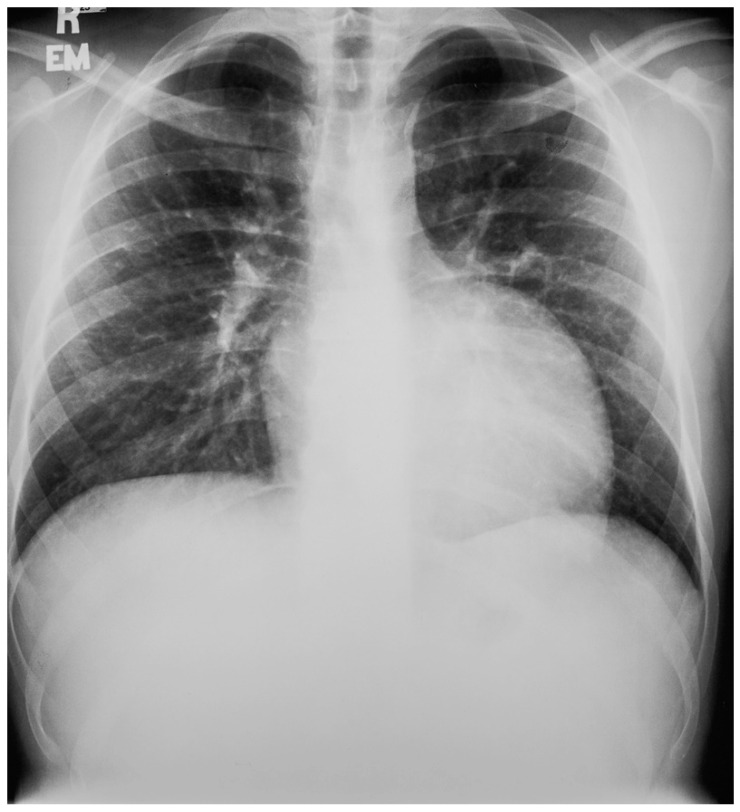
Posteroanterior chest X-ray in congenitally corrected transposition of the great arteries: This 20-year-old male presented with dyspnea on exertion. The image is well centered and there is a good inspiration. There is an increased cardiothoracic ratio, due to a pronounced left heart border attributed to an enlarged systemic ventricle. There is a double shadow on the right heart border due to left atrial enlargement. There is a narrow waist between the great arteries and the heart that is made more evident by the enlarged cardiac silhouette. By palpation, the cardiac apex was laterally displaced. There was a grade 3/6 holosystolic murmur of systemic atrioventricular regurgitation at the cardiac apex. Doppler echocardiography demonstrated a markedly dilated systemic ventricle, with severe systemic atrioventricular regurgitation into a dilated left atrium consistent with congenitally corrected transposition of the great arteries; no other congenital cardiac anomaly was identified. Medical treatment for heart failure was initiated.

Congenitally corrected transposition of the great arteries (CCTGA) is also known as L-Transposition of the Great Arteries (L-TGA). It comprises less than 1% of congenital heart disease. This rare congenital heart defect is composed of atrioventricular discordance and ventriculoarterial discordance (Figure 11). These two discordances correct each other, hence the word corrected is included in the name [11]. In the presence of only one of these discordances cyanosis will result, but this cyanosis is corrected by the two discordances coinciding. In CCTGA the anatomic right ventricle serves as the systemic ventricle, a function it is not fit to perform. The thin-walled anatomic right ventricle dilates when facing systemic blood pressure and its tricuspid valve, the systemic atrioventricular valve, regurgitates into the left atrium. This leads to systemic ventricular failure. The anatomic left ventricle serves as the pulmonary ventricle. The pulmonary trunk in CCTGA is positioned to the right of the ascending aorta and the ascending aorta is to the left of normal. Together, these great artery malpositions account for their narrowed transverse dimension above the atria and ventricles on a PA CXR of CCTGA (Figure 12). Some patients with CCTGA may have variable position in the chest, with up to 20% having dextrocardia where the cardiac apex is pointing to the right.

### 2.8. Congenitally Corrected Transposition of the Great Arteries Presenting as an Adult after a Long Hiatus from Pediatric Care

#### Anatomy and Physiology of Congenitally Corrected Transposition of the Great Arteries

**Figure 13:**
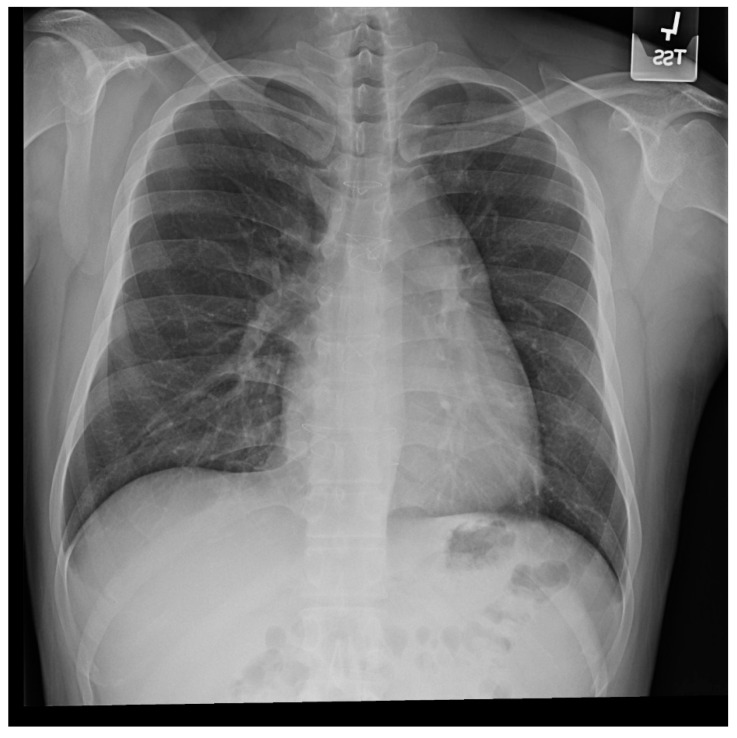
Posteroanterior chest X-ray in congenitally corrected transposition of the great arteries in adulthood: This is a PA chest X-ray of a 41-year-old male who was diagnosed with congenitally corrected transposition of the great arteries (CCTGA) shortly after birth. He underwent surgical closure of a large atrial septal defect (ASD) while still under the care of a pediatric cardiologist. He remained asymptomatic and regular care was not continued as an adult. When he developed rapid atrial fibrillation with reduced functional capacity he sought medical attention and completed a chest X-ray. The image is well centered and the inspiration is good. There is mesocardia where the cardiac apex is pointing midline. The cardiothoracic ratio is <0.5. The bifurcation of the trachea demonstrates an obtuse angle between the main bronchi. This suggests left atrial enlargement, which likely accounts for the onset of atrial fibrillation. The left hilum is larger than expected for CCTGA, but this might be related to the previously-repaired ASD. The pulmonary vascularity is normal.

**Figure 14:**
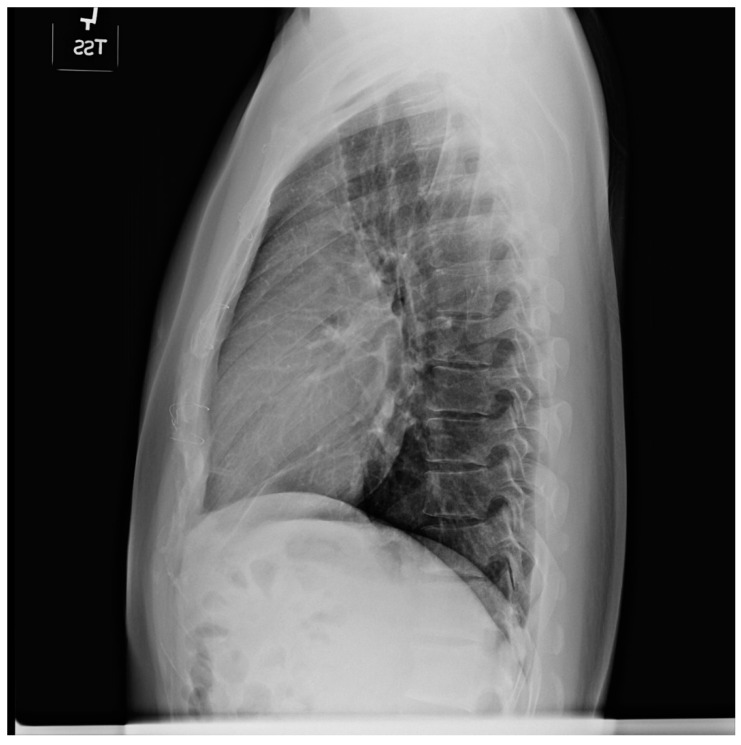
Lateral chest X-ray in congenitally corrected transposition of the great arteries in adulthood: this is the lateral chest X-ray of the same 41-year-old male in Figure 13. There are sternal wires from the median sternotomy used to repair his atrial septal defect, remote from this presentation. The retrosternal airspace is opacified, suggesting right ventricular enlargement. On examination he had a holosystolic murmur of atrioventricular valve regurgitation. Doppler echocardiography demonstrated severe systemic atrioventricular valve regurgitation (the anatomic tricuspid valve), and moderate dilatation of the systemic ventricle (the anatomic right ventricle) with preserved systolic function. He underwent replacement of the systemic atrioventricular valve with a dual-tilting disc mechanical prosthesis and did well. Optimally, the transition from pediatric congenital heart disease care to adult congenital heart disease care should be a coordinated hand-off [12].

The anatomy and physiology of CCTGA without additional congenital heart disease is described in the section above. In the majority of cases of CCTGA, other congenital structural cardiac anomalies coexist. These concomitant anomalies are ventricular septal defect (VSD), which occurs 70% of the time (usually perimembranous), an Ebstein-like systemic tricuspid valve, and pulmonary stenosis (PS), which occurs 40% of the time [7]. Figure 13 and Figure 14 showcase representative chest X-rays. The primary complications of adult patients with CCTGA are systemic atrioventricular valve regurgitation and complete atrioventricular electrical conduction block (third degree AV block).

### 2.9. Congenital Agenesis of the Right Pulmonary Artery

#### Anatomy and Physiology of Congenital Agenesis of the Right Pulmonary Artery

**Figure 15:**
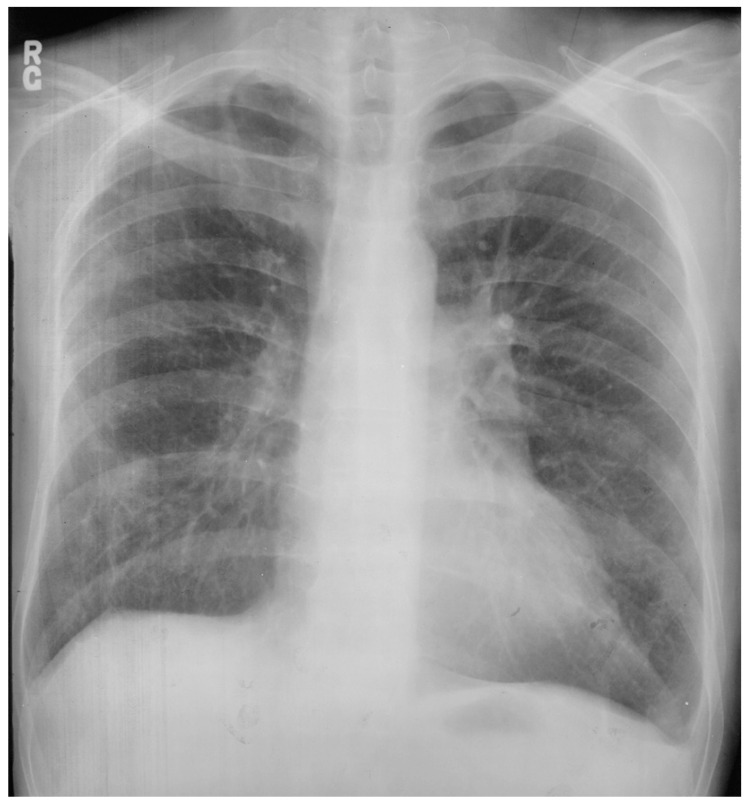
Posteroanterior chest X-ray of congenital absence of the right pulmonary artery: This is the chest X-ray of a 43-year-old male who sought another opinion because, after a sudden illness, he was dependent on supplemental oxygen. He was a coal miner who collapsed in the mine with chest pain and dyspnea one month before this CXR was done. When acutely ill he was taken to the hospital and found to have multiple pulmonary emboli to the lower left lung and no perfusion of the right lung. He was treated with intravenous heparin and warfarin. His chest pain resolved but he was not able to wean off the supplemental oxygen due to dyspnea at rest. This posteroanterior chest X-ray is well centered and there is a good inspiration. The cardiothoracic ratio is normal. The entire right lung is hypolucent with decreased vascularity in the left lower lung, compared to the left upper lung. There is pleural thickening at the left costophrenic angle. These left lung and left pleural findings are probably residua of the pulmonary emboli. Physical examination was not revealing. He underwent digital subtraction angiography with contrast injection into the right atrium. It showed congenital agenesis of the right pulmonary artery. The further compromise of congenitally halved pulmonary artery circulation by the pulmonary emboli rendered this man hypoxic and dyspneic. He was encouraged that due to the good medical care he received, he survived a life-threatening illness. Now that he requires supplemental oxygen he can no longer work in the coal mine.

Unilateral pulmonary artery agenesis (UPAA) is a rare congenital anomaly that has minimal or no symptoms and is usually diagnosed in adulthood. When it is associated with other cardiovascular abnormalities it is usually diagnosed in childhood. In a study of military recruits in Greece over 4 years [13], 6 male patients, aged 17–20 years-of-age, were identified to have UPAA by a unilateral hyperlucent lung on PA CXR. Follow-up digital subtraction angiography made the diagnosis in all cases. The UPAA was on the left in four patients and on the right in two patients. In the absence of pulmonary arterial blood flow, the lung remains viable via bronchial arteries (most often branches of the descending aorta), although not serving hemoxygenation. The work capacity is challenged by only one lung receiving pulmonary arterial blood flow. Figure 15 is a posteroanterior CXR of congenital agenesis of the right pulmonary artery. 

### 2.10. Persistent Left Superior Vena Cava

#### Anatomy and Physiology of Persistent Left Superior Vena Cava

**Figure 16:**
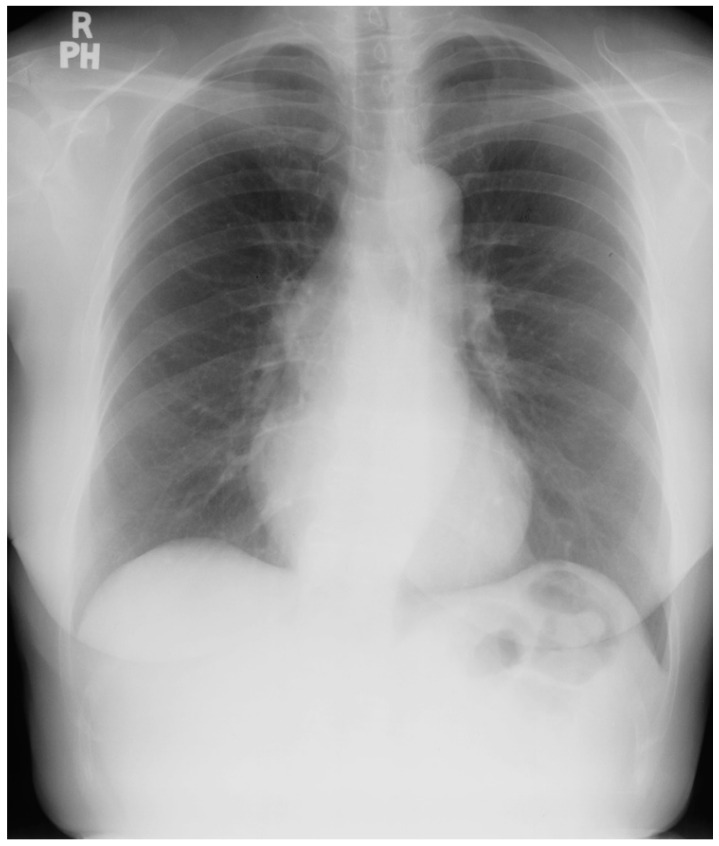
Posteroanterior chest X-ray of persistent left superior vena cava: This chest X-ray was obtained in an elderly female with angina pectoris who was preparing for coronary artery bypass surgery for ischemic heart disease. The image is rotated slightly to the left. There is a good inspiration and the cardiothoracic ratio is normal. The lung fields are clear and the pulmonary vascularity is normal. In this case of persistent left superior vena cava (PLSVC), the ascending aorta is more clearly imaged because the normal right-sided superior vena cava is not present to obscure the ascending aorta. Overlying the upper descending aorta and coursing parallel to the vertebral column and crossing the left main bronchus is a vascular structure that appears to be the PLSVC, prior to draining into the coronary sinus.

**Figure 17:**
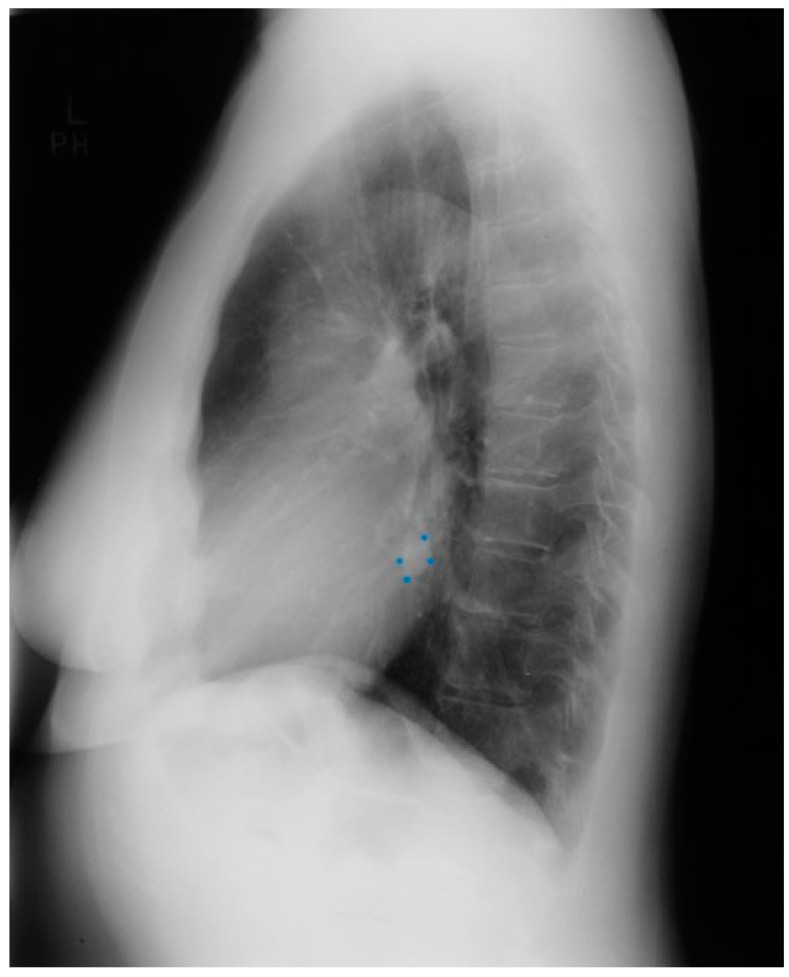
Lateral chest X-ray of persistent left superior vena cava: This is the lateral chest X-ray of the same patient with persistent left superior vena cava (PLSVC), whose images are discussed above. The most noteworthy finding is the round density located close to the posterior cardiac border, which is about equidistant from the leaves of the diaphragm and the radiolucent left main bronchus. This density (blue dots) is in the region of the posterior atrioventricular groove, where the coronary sinus is located. The coronary sinus is enlarged because, in addition to carrying coronary venous blood, it is carrying venous blood from the upper body, including the arms, head and neck, and chest. Thus, the posteroanterior and lateral chest X-rray can demonstrate findings that identify PLSVC. The anteroposterior chest X-ray done after coronary artery bypass graft surgery demonstrated passage of the right internal jugular venous introducer to the left side of the upper chest. The pulmonary artery catheter threaded through this introducer entered the PLSVC, then the coronary sinus to the right atrium, followed by the right ventricle and out to the pulmonary trunk. The patient recovered well from her bypass surgery.

Persistent Left Superior Vena Cava (PLSVC) is a congenital anomaly that results from failure of closure of the left anterior cardinal vein. In these patients, both right and left brachiocephalic veins drain into the PLSVC [14]. The PLSVC drains into the right atrium via the coronary sinus. PLSVC is the most common thoracic venous anomaly [15]. It is often diagnosed as an incidental finding when a CXR is done after the placement of a central venous catheter or pulmonary artery catheter. It can be suspected when the coronary sinus is found to be enlarged by echocardiography. When a dilated coronary sinus is noted, and PLSVC is suspected, injection of agitated saline individually into the right and the left antecubital veins is performed while imaging the heart with a somewhat posteriorly-angled apical four-chamber image. If there is a PLSVC, the enlarged coronary sinus is opacified first, followed by the right atrium. If the PLSVC is not associated with other congenital cardiac anomalies, it is usually asymptomatic and hemodynamically insignificant. An absent right superior vena cava can pose management difficulties during various cardiovascular procedures that involve accessing the heart via the superior vena cava, such as placement of a pacemaker wire, bicaval cannulation for cardiopulmonary bypass or venous cannulation for extracorporeal membrane oxygenation, among others. Figure 16 and Figure 17 represent CXRs of persistent left superior vena cava.

### 2.11. Ebstein Anomaly

#### Anatomy and Physiology of Ebstein Anomaly

**Figure 18:**
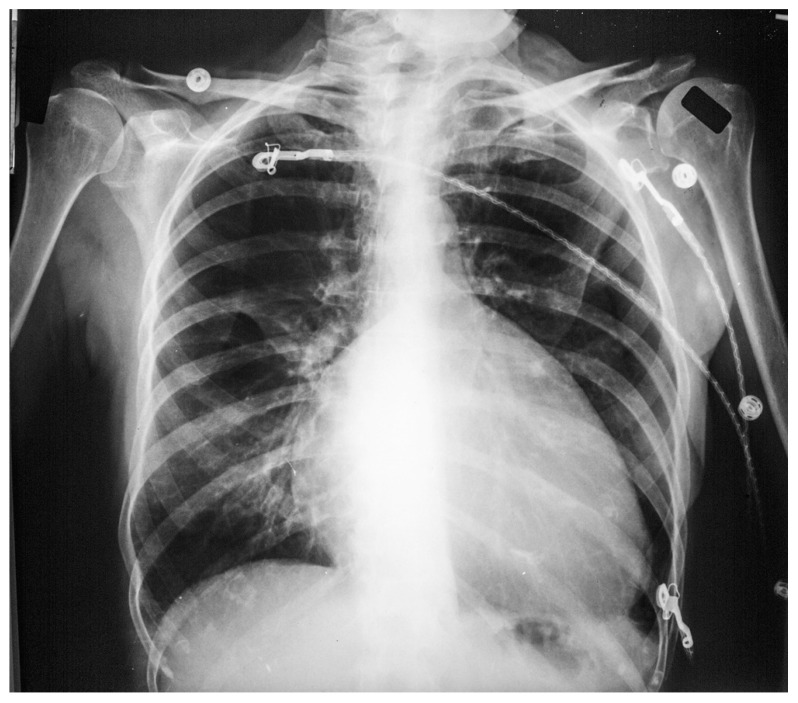
Anteroposterior chest X-ray of Ebstein Anomaly: This chest X-ray was obtained in a 73-year-old female who presented to the emergency department complaining of the abrupt onset of tachycardia. The chest X-ray is reasonably well centered and there is a good inspiration. There are three telemetry wires connected to the electrocardiogram electrodes, located on the right upper chest, left upper chest and left lower chest. Three metallic hospital gown snaps are evident. There is a globular heart with a markedly increased cardiothoracic ratio. Perhaps the cardiothoracic ratio would be smaller if this were a posteroanterior chest X-ray. The pulmonary vascularity is decreased and the aorta is small. The electrocardiogram showed atrial flutter with 2:1 conduction. Her rhythm spontaneously converted to sinus rhythm and the patient preferred further cardiac evaluation as an outpatient.

Ebstein anomaly (EA) involves anatomic and functional abnormalities of the tricuspid valve and right ventricle. EA is a rare congenital malformation that accounts for approximately 1% of all congenital defects of the heart [7]. The clinical presentation of native EA depends on the extent of deformity of the tricuspid valve, the size and function of the right ventricle, the size of the right atrium, the degree of tricuspid regurgitation, the presence and degree of valvular pulmonary stenosis, right atrial pressure and presence or absence of right-to-left shunt. Patients who first present as an adult with EA most commonly complain of dyspnea on exertion, fatigue, arrhythmias, and right heart failure. When EA is accompanied by an ASD or patent foramen ovale (PFO) the patient may be variably cyanotic, especially with exertion. Paradoxical emboli may result. Because EA is often accompanied by one or more accessory pathways and there is marked right atrial enlargement, supraventricular tachycardia should be anticipated in adults with EA. Other associated cardiac anomalies include ventricular septal defects and right ventricular outflow tract obstruction. Physical examination is different from patient to patient with EA. In the presence of an ASD or PFO central cyanosis may be present. Due to low cardiac output peripheral cyanosis may be present. Because the tricuspid valve is usually apically displaced into the right ventricle, the right atrium is large and compliant. Thus, despite the presence of severe tricuspid regurgitation the jugular venous pressure is often normal. A right parasternal lift may be palpated. Cardiac auscultation demonstrates a loud S1 and there may be one or more ejection clicks. The murmur of tricuspid regurgitation is holosystolic and located at the lower left sternal border. The CXR may be normal in mild cases of EA. In more severe cases there is marked C-T ratio enlargement (Figure 18). The right atrium is markedly enlarged and the cardiac contour is globular. The lung fields are clear and the pulmonary vascularity is decreased because the volume of blood pumped to the pulmonary arteries is decreased.

### 2.12. Severe Pulmonary Valve Stenosis

#### Anatomy and Physiology of Severe Pulmonary Valve Stenosis

**Figure 19:**
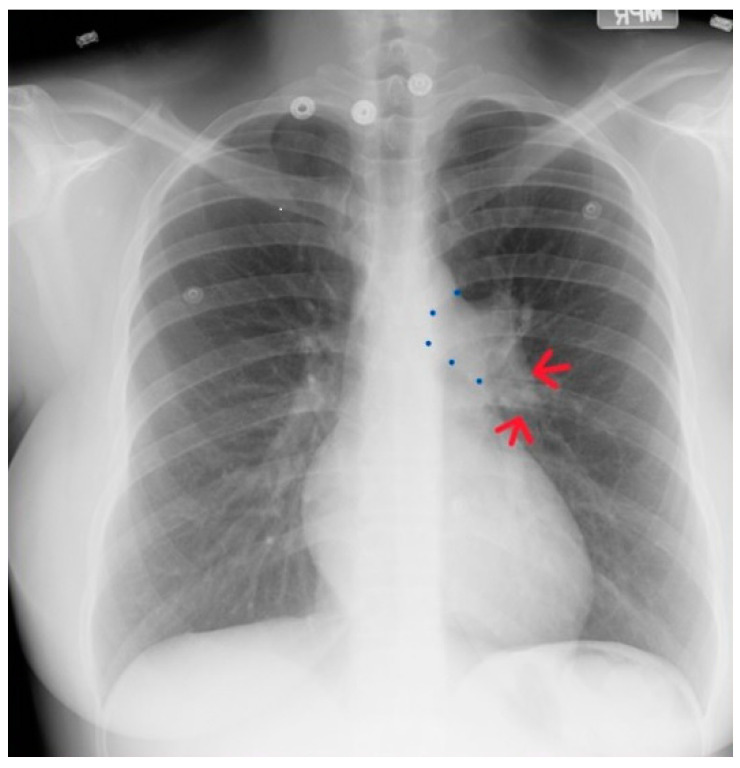
Posteroanterior chest X-ray of severe pulmonary stenosis: this chest X-ray is of a 31-year-old female who presented with worsening exertional dyspnea over the last two years. Her exam revealed elevated jugular venous pressure with prominent A wave. She was noted to have a right ventricular lift with a grade 4/6 systolic ejection murmur best heard at the left upper sternal border. The chest X-ray is well centered and there is a good inspiration. The cardiothoracic ratio is normal. The pulmonary trunk is enlarged (its right border is outlined by blue dots as it passes over the left bronchus). The left pulmonary artery branch that is indicated by two red arrows is also enlarged. Her echocardiogram revealed severe pulmonary valve stenosis with peak gradient 68 mmHg and mild pulmonary valve regurgitation. She subsequently underwent balloon valvuloplasty with reduction of her peak gradient to 24 mmHg. Her symptoms of dyspnea had resolved a few months post-procedure.

Valvular PS is usually an isolated lesion that occurs in 7–12% of all congenital heart disease cases [7]. The most common form of valvular PS features a dome-shaped pulmonary valve with a narrow central opening but a preserved, mobile valve mechanism. The pulmonary trunk is dilated and the jet from the stenotic valve favors flow to the left pulmonary artery branch. The PS gradient is categorized as mild when peak gradient is <36 mmHg, moderate when 36–64 mmHg, and severe when >64 mmHg. Valvular PS usually presents as an asymptomatic systolic murmur, but severe PS often features intolerance to exercise. Physical examination in a patient with severe PS is usually characterized by an elevated jugular venous pressure with a prominent A wave, an RV heave, and a long, loud systolic flow murmur with wide splitting of S2, if the P2 component is audible. In a study of 87 children aged 4–14 years-of-age with valvular pulmonary stenosis proven by cardiac catheterization, CXR evidence of poststenotic dilatation of the pulmonary trunk (Figure 19) and the left pulmonary artery branch, together with a normal or increased left- sided perihilar pulmonary vascularity, was essential for the diagnosis of valvular pulmonary stenosis [16]. These CXR findings of PS increased with age of the child, but no correlation between these radiographic observations and the severity of the stenosis was found. These same CXR findings are noted in the adult with valvular PS.

### 2.13. Marfan Syndrome

Marfan syndrome (MFS) is an autosomal dominant, highly penetrant condition that is caused by pathogenic variants in FBN1, which encodes fibrillin-1, a major structural component of extracellular matrix in connective tissues [17]. The major manifestations of MFS are asymptomatic aortic root aneurysms, aortic dissections, dislocation of the ocular lens (ectopia lentis) and skeletal abnormalities which include overgrowth of long bones and thoracic cage deformities. It is important to know these major manifestations in order to suspect the presence of MFS in a patient who has no symptoms. This can be done by inspection or by learning a family history of sudden death due to aortic rupture. 

#### Anatomy and Physiology of Marfan Syndrome

**Figure 20:**
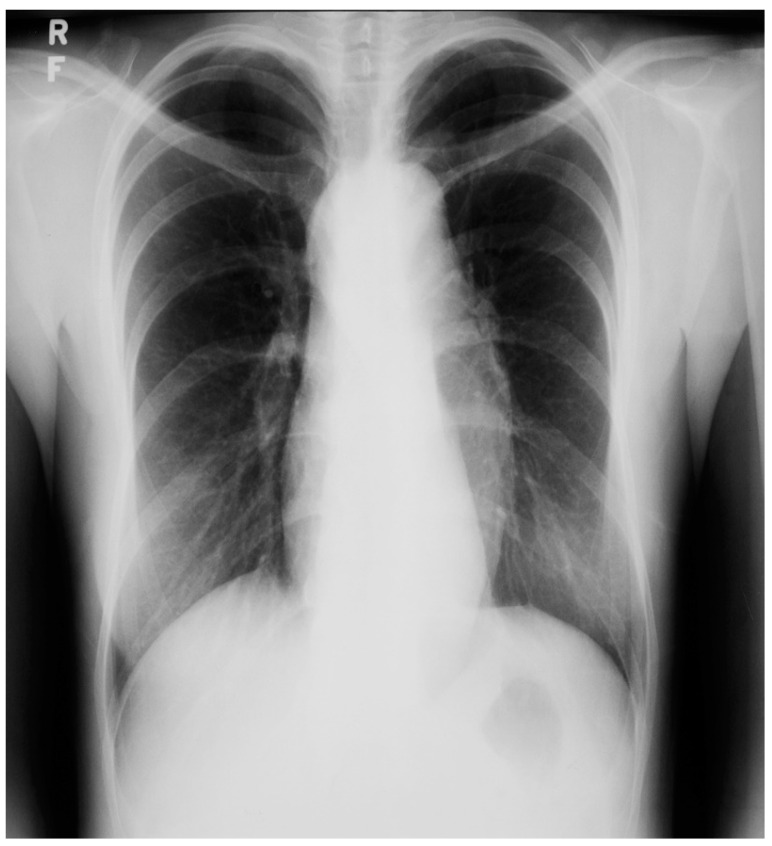
Posteroanterior chest X-ray of Marfan syndrome: This 24-year-old female with Marfan syndrome had a chest X-ray because of her tall, thin stature and hyperextensible joints. She was asymptomatic. The image is well centered and the inspiration is good. Notice the widened upper posterior dorsal intercostal spaces. This indicates an increased dorsal kyphosis. The C-T ratio is normal. The ascending aorta, aortic arch and descending aorta are dilated. There was a faint decrescendo diastolic murmur of aortic regurgitation at the right upper sternal border. The normal heart size suggests that, in this asymptomatic woman, the aortic regurgitation was mild.

**Figure 21:**
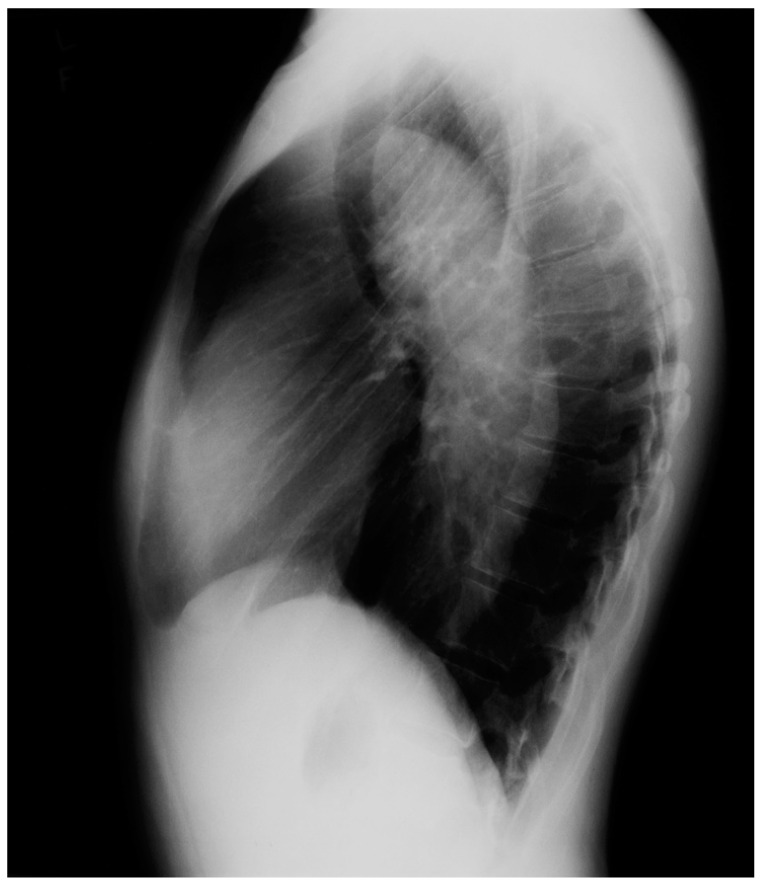
Lateral chest X-ray of Marfan syndrome: this chest X-ray was done on the same 24-year-old female above. It shows the dorsal kyphosis and pectus carinatum (also known as pigeon chest). The ascending, arch, and descending aorta were markedly dilated. Additional imaging measured the minimum thoracic aortic diameter to be 6 cm. She went on to have a mechanical aortic valve replacement, followed by aortic root and ascending aortic replacement via median sternotomy, and then descending aortic replacement via left lateral thoracotomy. A few years later she required an extensive abdominal aortic replacement with multiple arterial anastomoses to vital organs supplied by the abdominal aorta. She was followed for 30 years after her first diagnosis of MFS and her first aortic operation and remained NYHA functional class 1–2. Suspicion of the diagnosis of MFS in an asymptomatic patient that leads to a CXR is an important first step in the optimal care of MFS.

In the majority of patients with MFS, thoracic aortic disease starts with enlargement of the aortic root and progressively enlarges to form an aneurysm (Figure 20 and Figure 21). This is the most life-threatening manifestation of MFS. Since medications can retard aortic enlargement, early identification affords optimal care. While long limbs and hyperextensible joints can be assessed by inspection, the CXR can assess thoracic cage deformities and aortic size. 

### 2.14. Tetralogy of Fallot (toF)

#### Anatomy and Physiology of Tetralogy of Fallot

**Figure 22:**
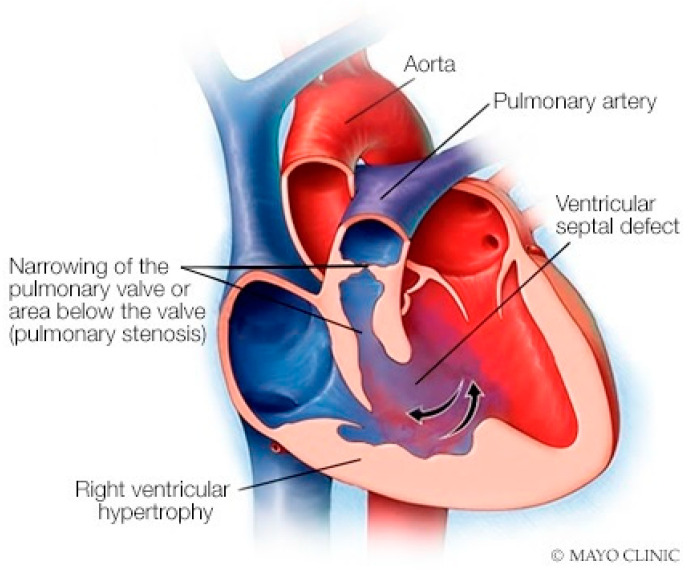
Tetralogy of Fallot: The four components of tetralogy of Fallot include a ventricular septal defect, an over-riding aorta, right ventricular hypertrophy and right ventricular outflow tract obstruction, which may be subpulmonary and/or at the pulmonary valve level.

**Figure 23:**
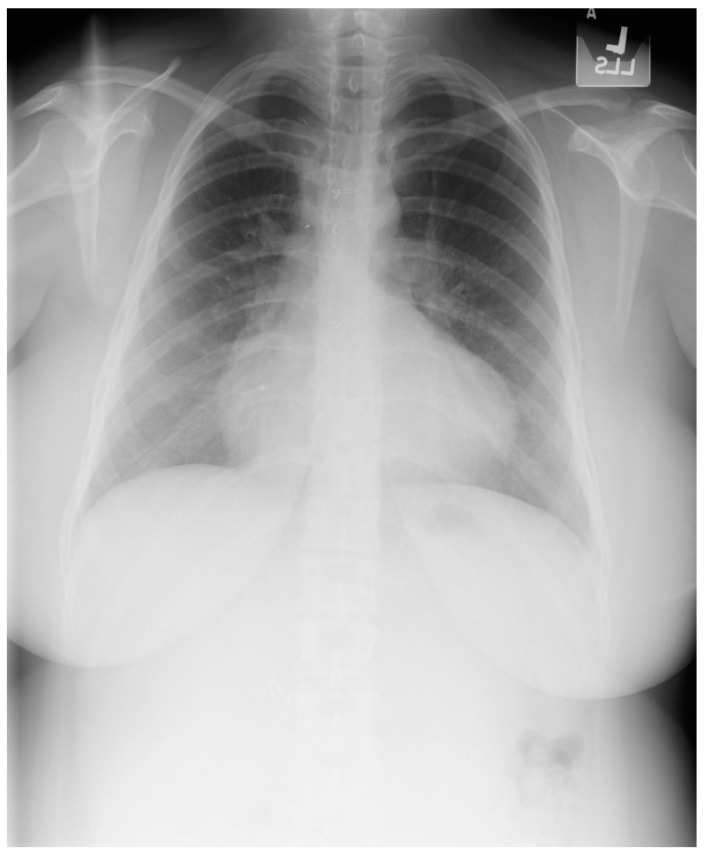
Posteroanterior chest X-ray in an adult with previously operated tetralogy of Fallot: the chest X-ray was obtained in a 21-year-old female with exertional dyspnea. She was cyanotic at birth and diagnosed with tetralogy of Fallot. She had a Blalock-Thomas-Taussig (BTT) shunt (palliative subclavian artery to pulmonary artery shunt) at 10-months-of-age. At 3-years-old she underwent complete repair with closure of the ventricular septal defect, right ventricular (RV) outflow tract reconstruction with pericardium, with transannular patch and ligation of the BTT shunt. This posteroanterior chest X-ray is well centered and the inspiration is good. The cardiothoracic ratio is increased and the cardiac silhouette is boot-shaped (“coeur en sabot”). The right heart border is prominent, suggesting right atrial enlargement. The pulmonary vascularity is normal and the lung fields and pleura are clear.

**Figure 24:**
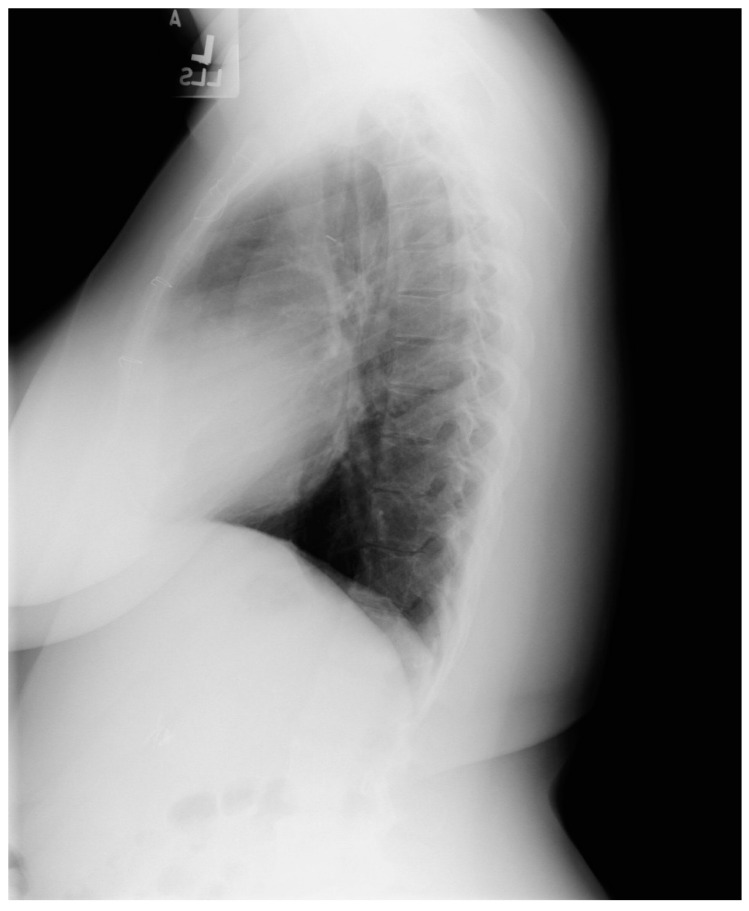
Lateral chest X-ray in an adult with previously operated tetralogy of Fallot (toF) above: This X-ray shows sternal wires from a previous median sternotomy. The retrosternal airspace is mildly occupied by right ventricular enlargement. There is a metallic surgical clip in the upper middle mediastinum from one of her previous surgical procedures. Doppler echocardiography revealed severe pulmonary valve regurgitation, moderate–severe tricuspid regurgitation, moderate–severe right ventricular dilation and dysfunction. The patient subsequently underwent pulmonary valve replacement and tricuspid valve repair.

Tetralogy of Fallot (toF) is the most common cyanotic congenital heart disease in infants. Its four components include subpulmonary infundibular stenosis, a ventricular septal defect (VSD), an overlying aorta that straddles the VSD, and right ventricular hypertrophy (Figure 22), because the workload of the right ventricle is essentially at systemic pressure. As an infant and child, patients with toF are functionally limited, and are commonly treated surgically to palliate or to repair the defects. Accordingly, most children with toF will live well into adulthood [18]. These adult patients with toF are among the most commonly seen by cardiologists who practice ACHD, but these patients might also visit a general cardiologist or a general physician. One of the most common problems encountered by a patient late after toF repair is progressive dilatation of the right ventricle [12] in the setting of important pulmonary valvular regurgitation. Other less common complications include arrhythmias, aortopathy, tricuspid or aortic valve regurgitation, ventricular septal patch leak, and left ventricular systolic dysfunction. It is essential for the physician who follows an adult patient with toF that was repaired as a child, who develops progressive right ventricular dilatation, to recognize that pulmonary valve regurgitation is the usual cause and it is remediable. Uncorrected, this progressive right ventricular enlargement can lead to potentially lethal ventricular arrhythmias. Figure 23 and Figure 24 represent PA and lateral CXR of repaired toF.

### 2.15. Patent Ductus Arteriosus with Eisenmenger Syndrome

#### Anatomy and Physiology of Patent Ductus Arteriosus with Eisenmenger Syndrome

**Figure 25:**
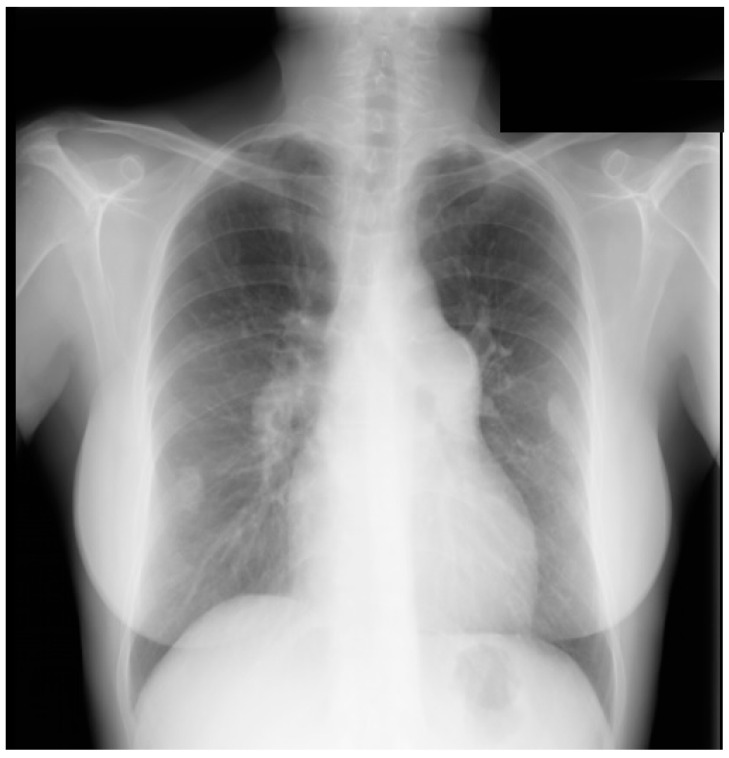
Posteroanterior chest X-ray of patent ductus arteriosus with Eisenmenger syndrome: this X-ray was obtained in a 56-year-old dyspneic female with Eisenmenger syndrome from an unrepaired patent ductus arteriosus (PDA). She was born in 1948 with a murmur noted at birth, at a time when no surgical intervention was possible. She was restricted from physical activity as a child and started to notice exertional dyspnea in her late teens. Cardiac catheterization at the time led to the diagnosis of Eisenmenger syndrome with PDA. This posteroanterior chest X-ray is well centered and there is a good inspiration. The cardiothoracic ratio is <0.5. The pulmonary trunk is enlarged, with calcification noted in its lateral perimeter. There is pruning of the peripheral pulmonary arteries. There appears to be exuberant callus formation at healed fractures of the right posterior eighth rib and the left posterior seventh rib.

**Figure 26:**
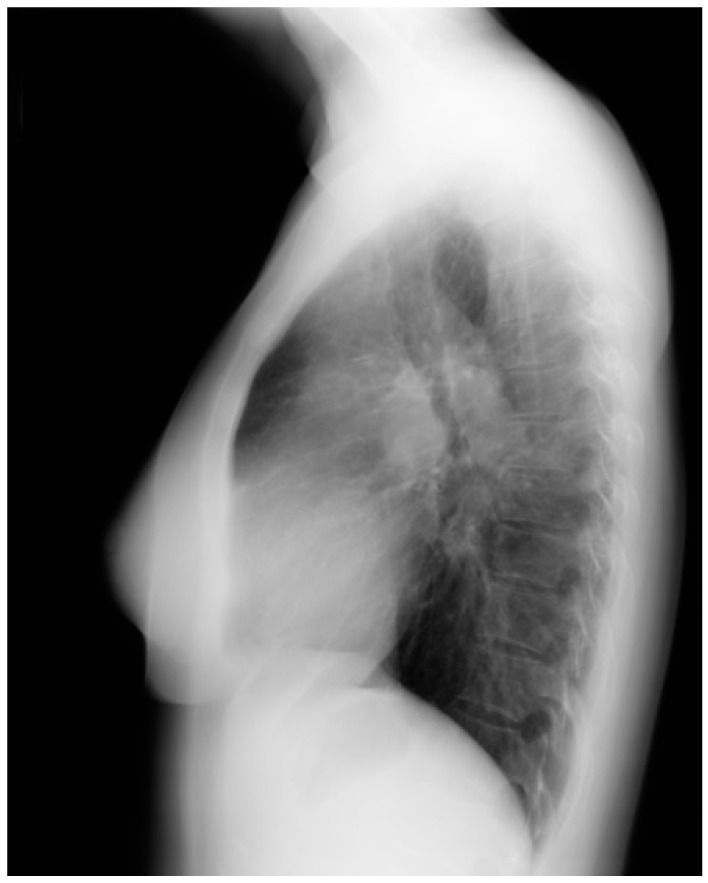
Lateral chest X-ray of patent ductus arteriosus with Eisenmenger syndrome: This is the lateral chest X-ray of the same 56-yo F with an unoperated patent ductus arteriosus (PDA) and Eisenmenger syndrome. The central pulmonary arteries are enlarged. The retrosternal airspace is encroached upon by right ventricular (RV) enlargement. Doppler echocardiography demonstrated a calcified PDA with mild right-to-left shunting, moderate RV dilatation and systolic dysfunction with severe RV hypertrophy. There was moderate pulmonary regurgitation and moderate tricuspid regurgitation. Clinically, she was NYHA Functional Class 2.

Patent Ductus Arteriosus (PDA) is a persistent communication between the aorta and the pulmonary artery, long after this vital fetal connection should have closed a few days after birth. Unoperated patients may present with a heart murmur or symptoms of shortness of breath and easy fatigue. A patient with a large and unrestricted PDA may present with Eisenmenger physiology with differential cyanosis (cyanosis of the lower extremities with normal upper extremity nailbeds) and clubbing [7]. The murmur of a moderate or large PDA is a continuous machinery murmur. In the presence of pulmonary artery hypertension (PAH) only a systolic murmur is heard. In the presence of a PDA with a large left-to-right shunt the blood pressure has a wide pulse pressure. When there is PAH from a PDA with right-to-left shunting at ductal level, lower extremity clubbing and cyanosis is found. Be sure to examine the feet. The CXR in PDA depends on the size of the shunt. A prominent proximal pulmonary artery segment indicates elevated pulmonary artery pressure (Figure 25 and Figure 26). An enlarged left atrium and enlarged left ventricle indicate a significant PDA with left-to-right shunting. Calcification in the region of the ductus should be noted because it may rupture during surgical repair [7]. Eisenmenger syndrome typically appears later in life, but patients with a large PDA are among the earliest congenital heart disease patients to present with Eisenmenger syndrome. These patients require close follow-up. Due to a high maternal mortality, pregnancy is not recommended in the presence of Eisenmenger syndrome [7]. Surgery with general anesthesia must be conducted in patients with Eisenmenger syndrome only in centers with expertise, as a fall in systemic pressure with induction of anesthesia might exacerbate the right-to-left shunt and cause prolonged systemic hypoxia, and potentially lethal vital organ injury.

## 3. Conclusions

Congenital heart disease (CHD) is composed of a broad spectrum of anatomic deformities. The anatomy and physiology of each congenital heart disease must be understood so that it can be corroborated with a patient’s complaints and physical examination to make a diagnosis. The CXR is a pictorial demonstration of cardiac, great vessel, and pulmonary anatomy and physiology. Thus, the CXR is like a memorable figure that clarifies what is described in the caption. Recall how rib notching can lead to the diagnosis of coarctation of the aorta, and how increased pulmonary vascularity can lead to a diagnosis of a left-to-right shunt. In additional to findings of the clinic exam, the diagnosis of ACHD can be further facilitated with echocardiography and tomographic imaging. ACHD deals with making a diagnosis of CHD that was not recognized until adulthood, and with following the course of patients with CHD who were diagnosed, and possibly surgically treated, at a young age. Knowledge of potential postoperative complications is required in order to recognize these complications on serial CXRs as a patient ages with CHD. Recall severe pulmonary valve regurgitation with right ventricular enlargement leading to malignant ventricular arrhythmias in the adult patient who underwent complete repair of tetralogy of Fallot as a child. Clinicians who know CXRs in ACHD will carry the sharpest arrow in their quiver, and use it to hit the target and take the best care of their patients. 

## Data Availability

Not applicable.
